# Study of Uremic Toxin Fluxes Across Nanofabricated Hemodialysis Membranes Using Irreversible Thermodynamics

**DOI:** 10.5936/csbj.201303005

**Published:** 2013-06-11

**Authors:** Assem Hedayat, Rob Peace, Hamdi Elmoselhi, Ahmed Shoker

**Affiliations:** a College of Dentistry, University of Saskatchewan, 105 Wiggins Road, Saskatoon, SK, S7N 5E4, Canada; b Department of Mechanical Engineering, University of Saskatchewan, 57 Campus Drive, Saskatoon, SK, S7N 5A9, Canada; c Saskatchewan Transplant Program, St. Paul's Hospital, 1702, 20th Street West, Saskatoon, SK, S7M 0Z9, Canada; d Division of Nephrology, Department of Medicine, University of Saskatchewan, 103 Hospital Drive, Saskatoon, SK, S7N 0W8, Canada

**Keywords:** uremic toxins, nanofabrication, hemodialysis membranes, endothelin, cystatin C, interleukin-6

## Abstract

**Introduction:**

The flux of uremic toxin middle molecules through currently used hemodialysis membranes is suboptimal, mainly because of the membranes’ pore architecture.

**Aim:**

Identifying the modifiable sieving parameters that can be improved by nanotechnology to enhance fluxes of uremic toxins across the walls of dialyzers’ capillaries.

**Methods:**

We determined the maximal dimensions of endothelin, cystatin C, and interleukin – 6 using the macromolecular modeling software, COOT. We also applied the expanded Nernst-Plank equation to calculate the changes in the overall flux as a function of increased electro-migration and pH of the respective molecules.

**Results:**

In a high flux hemodialyzer, the effective diffusivities of endothelin, cystatin C, and interleukin – 6 are 15.00 x 10^-10^ cm^2^/s, 7.7 x 10^-10^ cm^2^/s, and 5.4 x 10^-10^ cm^2^/s, respectively, through the capillaries’ walls. In a nanofabricated membrane, the effective diffusivities of endothelin, cystatin C, and interleukin – 6 are 13.87 x 10^-7^ cm^2^/s, 5.73 x 10^-7^ cm^2^/s, and 3.45 x 10^-7^ cm^2^/s, respectively, through a nanofabricated membrane. Theoretical modeling showed that a 96% reduction in the membrane's thickness and the application of an electric potential of 10 mV across the membrane could enhance the flux of endothelin, cystatin C, and interleukin - 6 by a factor of 25. A ΔpH of 0.07 altered the fluxes minimally.

**Conclusions:**

Nanofabricated hemodialysis membranes with a reduced thickness and an applied electric potential can enhance the effective diffusivity and electro-migration flux of the respective uremic toxins by 3 orders of magnitude as compared to those passing through the high flux hemodialyzer.

## Introduction

High-flux hemodialyzers proved to be more efficient than cellulose membranes in removing middle molecules uremic toxins, and reducing the dialysis session by as much as 25% [1]. However, with its current state of the art technology, hemodialysis remains a suboptimal form of renal replacement therapy because it does not comprehensively simulate the continuous, selective, efficient filtration process of the kidneys. Nanotechnology may be the future venue to construct hemodialyzers that can mimic kidney function [2]. In this study, we selected a high-flux hemodialyzer, and characterized its structural and functional limitations to the sieving of middle molecules. The high flux hemodialyser we used is a hemodialysis filter built on the concept of hollow fiber filtration. Each of the 12,000 hollow fibers inside the dialyzer has a 215 µm inner diameter, and a 50 µm wall thickness. The walls have a three layers structure of a blend of polymeric materials. This layered architecture with increased porosity from the inner walls outward is to enhance diffusivity and filtration of the toxins through them [3]. The structure is based on a capillary design, where the blood passes through the capillaries, and the toxins pass through the capillary walls. The transported toxins are flushed by a countercurrent dialysate. Our objective was to characterize the morphologies of the outer, cross-sectional, and inner surfaces of the hollow fibers as a first step to identify their weaknesses. Identifying the modifiable sieving parameters led us to consider nanofabrication as a venue that has great potential to improve theses structural weaknesses.

The advantages of nanofabricated hemodialysis membranes as we envision can be summarized as follows:

- Improved pore density on the inner surface in contact with the blood
- Improved, non-tortuous channel structure
- Improved surface area / volume ratio
- Improved selective removal of uremic toxins
- Higher efficiency of removing uremic toxins
- Synergy between the driving forces of molecular sieving
- Relatively low ratio of the length of the straight transport path to the maximum dimension of the uremic toxins molecules
- Improved channel structure can produce more accurate kinetic models that can explain the transport mechanism inside the membrane and the sieving process
- Fluxes attributed to surface electric potential and other driving forces of filtration can be predicted with high precision and reliability using irreversible thermodynamic (non-equilibrium) modeling [4]

In this research, we applied irreversible (nonequilibrium) thermodynamic (IT) to model the contribution of specific gradients such us the electric potential and pH on the fluxes of middle molecules such as endothelin, cystatin C, and interleukin – 6 across nanofabricated membranes. Irreversible thermodynamics is the science that describes the increase in entropy of a species and the free energy dissipated as a result of it during a spontaneous, continuous process [5]. The species can be molecules mixed in a fluid that is subjected to a gradient. The gradient can be thermal, electrical or due to pressure or concentration for example. IT fits the process of hemodialysis well because there is a state of continuous nonequilibrium that drives the transport. It has numerous advantages over kinetic modeling [5, 6] amongst which is that in IT models, the filter is treated as a black box with no need to know the process involved in transporting the solute through it. It is also much less complex than kinetics models that involve solving complex differential equations within the membrane, and extensive experimentation to determine numerical coefficients that are sensitive to the driving forces of the sieving process [5]. IT is a very powerful tool to characterize and model the transport of uremic toxins across nanofabricated molecules.

## Methods

The samples were prepared for viewing under the scanning electron microscope (SEM) as detailed in our previous publication [3].

Uremic toxins molecules such as endothelin, cystatin C, and interleukin - 6 molecules should pass through the inner surface's pores. In this study, we were trying to delineate the relationship between the pore shape and molecular hindrance. The uremic toxin molecules have different shapes that limit their clearance. Accordingly, the shapes of the selected uremic toxins as well as others were studied to determine how current membranes handle their passage, and to help design nanofabricated novel membranes to optimize their clearance.

The Crystallographic Object-Oriented Toolkit (COOT) software [7] is very efficient in measuring the maximum dimensions of selected middle molecules such as endothelin, cystatin C, retinol-binding protein, complement factor D, interleukin-6, and Interleukin – 1 β. The Access codes for each of these molecules was resourced from the Protein Data Bank (PDB). The molecule was rotated in 3-dimensions, and the maximum linear dimension at each frame was determined. At least ten measurements were made for each molecule and the maximum dimension among all frames was recorded. The maximal molecular dimension with the narrowest pore width on the inner surface of the capillary was compared.

The objective of this study, as its title indicates, is to study uremic toxin fluxes across nanofabricated hemodialysis membranes using irreversible thermodynamics. Accordingly, irreversible thermodynamic modeling was the tool of preference to estimate the contribution of specific forces responsible for the transport of the middle molecules across nanofabricated membranes. In a previous publication [4] the Nernst-Plank equation was expanded to include the Kedem-Katchalsky equation [8], and the pH, as follows:

$$J=-D_{eff}AK_{diff}(\frac{dc}{dx})-\left(\frac{D_{eff}AC_{m}zF}{RT}\right)\left(\frac{dV}{dx}\right)-\left(\frac{D_{eff}AC_{m}}{x}\right)(-4.606pH-2.303\text{log}p_{H_{2}})+K_{conv}AC_{m}J_{v}+\Omega L_{p}A(\Delta P+\Delta \pi )$$

Previously, the different segments of the equation were used to theoretically estimate the fluxes of creatinine, β_2_-microglobulin, and tumor necrosis factor - α across nanofabricated membranes as a function of applied electric potential and changing pH at a reduced membrane thickness [4].

In this research, the contribution of an applied electric potential of 10 to 80 mV to the flux of three other uremic toxins: endothelin, cystatin C, and interleukin – 6 was estimated. The effect of change in pH on the flux of the respective molecules was also determined. Both effects were computed at a reduced thickness of the membrane of 1 micron.

The free diffusivity of the molecules (D_0_) through a nanofabricated membrane was calculated [9, 10] using the following equation:

$${\text{D}}_{0}=\frac{13.26\times 10^{-5}}{V_{M}^{0.589}n^{1.4}}$$

where η is water's viscosity at 37°C and V_M_ is the molecular volume calculated through classical crystallographic methods.

Using D_o_, D_eff_ can be determined from the equation:

$${\text{D}}_{\text{eff}}={\text{K}}_{\text{diff}}{\text{D}}_{0}$$

where, the diffusion hindrance, K_diff_, is calculated [11, 12] as follows:

$${\text{K}}_{\text{diff}}=1.0-2.3\lambda +1.154\lambda^{2}+0.224\lambda^{3}$$

where λ = r / b,

and b is the pore radius that we set at 10 nm and r is the molecules’ maximum dimension as estimated using COOT.

The effective diffusivities of the respective molecules were calculated using equation (3). The effective diffusivities through both the high flux hemodialyzer and a nanofabricated membrane were compared.

In case of nanofabricated membranes, the sieving coefficient, S, was considered to be equal to one. Since the concentration of endothelin, cystatin C, and interleukin-6 varies from one end stage renal disease patient to another, the normal concentration, C_m_, of these molecules in the blood was used as a reference. The normal concentrations of these molecules are listed in Table 1. The calculations are limited by using normal concentrations. However, further calculations will be necessary after obtaining consistent concentrations of these molecules in uremic patients.

**Table 1:** Normal concentration of selected uremic toxins in the blood [13].

Molecule	Normal Concentration
Endothelin	28.8 ± 3.8 ng/L
Cystatin C	< 1.6 mg/L
Interleukin - 6	13.3 ± 3.1 ng/L

Theoretical calculations using the normal concentrations were based on a cylindrical pattern applied to a nanofabricated membrane with the following suggested parameters: 10 nm pore radius, membrane thickness of 1 micron, and an overall membrane area of 1 m^2^. The K_diff_ for the nanofabricated membrane was estimated using equation (4).

Expanding the Nernst-Plank equation as given in equation (1) allowed us to estimate the contribution of the different forces involved in the filtration process on the total Flux. For example, the second term in equation (1), which represents J_electromigr_ for endothelin, cystatin C, and interleukin – 6 across nanofabricated membranes was calculated for two membrane thicknesses, 25 µm and 1 micron. Also, the third term in equation (1), which represents J_pH_, was calculated for the same molecules and for the same membrane thicknesses.

For the high flux hemodialyzer, the hindrance factor, K_diff_, was calculated [14] using the equation:

$${\text{K}}_{\text{diff}}={\left(\varepsilon /(2-\varepsilon )\right)}^{2}$$

where ɛ is the open area estimated at 5.45%.

## Results

### Morphological characterization of the high flux hemodialyzer

**Figure 1:**
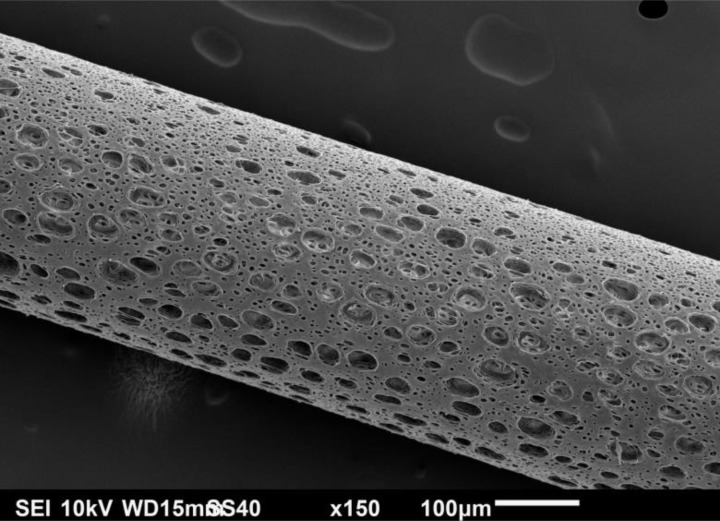
SEM photomicrograph of a capillary extracted from a high flux hemodialysis filter.

**Figure 2:**
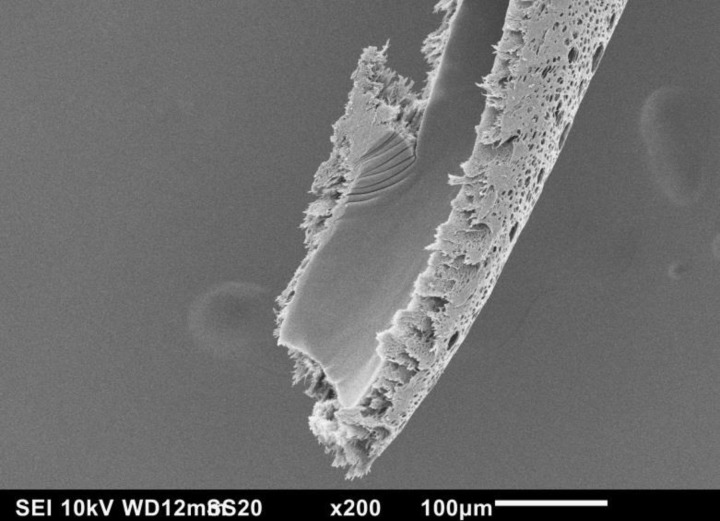
SEM photomicrograph of a longitudinal cross-section of a high flux hemodialysis capillary.

SEM photomicrographs reveal detailed structural features of the inner and outer surfaces of the high flux hemodialyzer's capillary. Figure 1 shows the fenestrations on the outer surface of the capillary. Slicing of the capillary longitudinally revealed a vast variation between the morphologies of both of the outer and inner surfaces of the capillary, as shown in Figure 2. At a 200 x magnification, the fenestrations are visible on the outer surface, while in contrast, the inner skin surface of the capillary is much smoother, and no fenestrations are visible at this magnification.

### Determining the functional / structural limitations of current hemodialysis membranes

**Figure 3:**
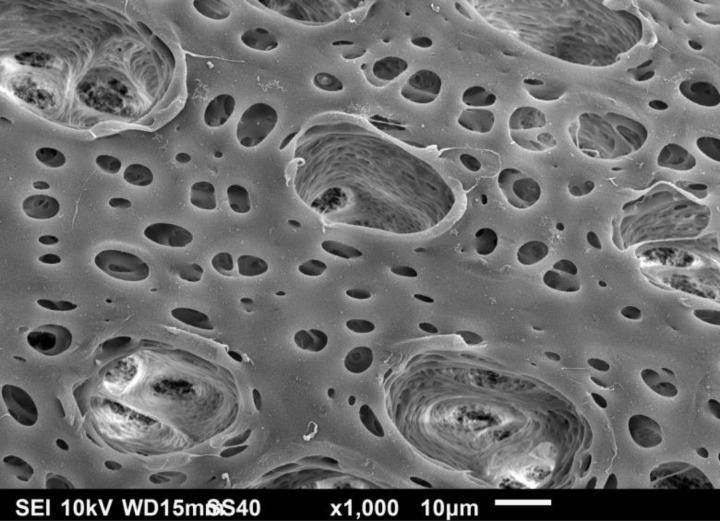
SEM photomicrograph of the open pores in contact with the dialysate.

Figure 3 is a closer look at the open pores on the surface of the capillary that are in contact with the dialysate. Slicing of the capillaries transversely yielded a columnar structure extending from the inner skin layer of the capillary and its encapsulating thin spongy structure to the outer surface of the capillary. This finger like structure provides mechanical support to the whole capillary's architecture. It is also noticeable, how the porosity increases from the skin layer through the spongy layer, and towards the capillary's perimeter. This layered structure is similar to the one observed in another hemodialyzer hollow fiber [15].

**Figure 4:**
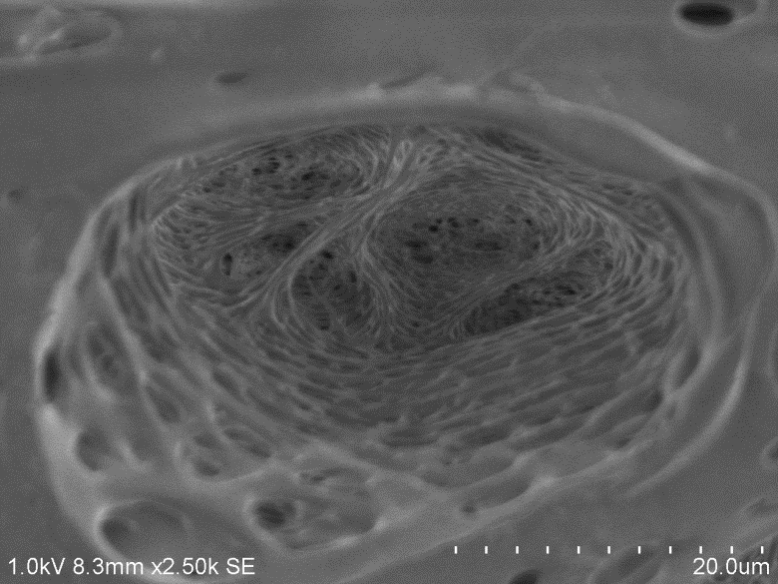
Morphological characterization of the interior of on outside pore that lies on the circumference of a high flux hemodialysis capillary.

Examination of the inner surface of the capillary reveals very fine fenestrations. It is interesting to note that the ratio of the mean width of an outer surface pore to an inner surface one is about 917 times, which is a measure of increased porosity radially towards the outer surface of the capillary.Characterization of the interior of the outer surface pores using the FESEM reveals a very tortuous and complex network of channels through which the uremic toxin molecule has to travel before being flushed by the dialysate. This is illustrated in Figure 4.

### Identifying the modifiable sieving parameters that can be improved by nanotechnology to enhance fluxes of uremic toxins across the walls of dialyzers’ capillaries

**Figure 5:**
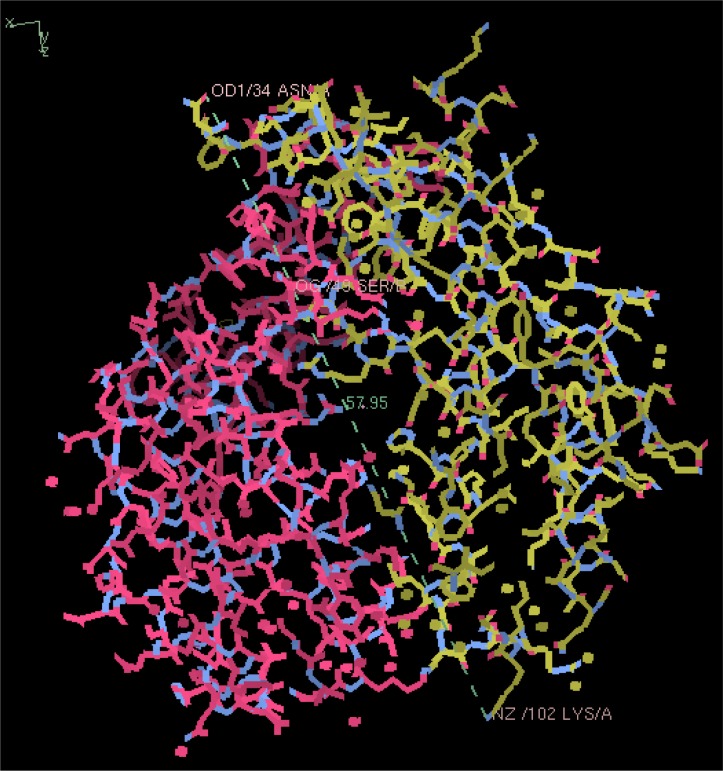
Measurement taken on a tumor necrosis factor – α molecule using the Protein Data Bank accession code: 3GIO.

Using COOT, the maximum dimension for the uremic toxins smaller than albumin were determined to be less than the narrowest pore on the inner surface of the capillary by at least 3 folds. Figure 5 is a sample of the measurement taken on a tumor necrosis factor – α molecule. The farthest two points in the molecule as it was rotated in 3-D yielded 57.95 Å, which is 5.795 nm. Table 2. summarizes the molecular maximum dimensions of selected uremic toxins and lists their Protein Data Bank Access Codes [16].

**Table 2:** Maximum dimension of selected uremic toxins measured using COOT software.

Uremic Toxin	PDB Access Code	Maximum Dimension (nm)
Endothelin	1EDN	2.6
Cystatin C	3GAX	4.08
Retinol-binding protein	1BRP	4.9
Complement Factor D	1DSU	5.12
Interleukin-6	1ALU	5.18
Tumor necrosis factor – α	3GIO	5.79
Interleukin – 1 β	3LTQ	6.04

### Utilizing irreversible (non-equilibrium) thermodynamics to model the enhancement of uremic toxins transport in nanofabricated hemodialysis membranes

A comparison between the effective diffusivities of the selected uremic toxins in the high flux hemodialyzer and a nanofabricated membrane is given in Table 3. It shows that the diffusivity of the respective molecules passing through the nanofabricated membrane 1 micron thick is three orders of magnitude greater than that passing through the walls of a high flux hemodialyzer's hollow fiber.

**Table 3:** Effective Diffusivity (D_eff_) of Selected Uremic Toxins Through a High Flux Hemodialyzer and a Nanofabricated Membrane.

Uremic Toxin	D_eff_ in a High Flux Hemodialyzer (cm^2^/s)	D_eff_ in a Nanofabricated Membrane (cm^2^/s)
Endothelin	15 x 10^-10^	13.87 x 10^-7^
Cystatin C	7.7 x 10^-10^	5.73 x 10^-7^
Interleukin-6	5.4 x 10^-10^	3.45 x 10^-7^

For the 1 micron thick membrane, the fluxes for each respective molecule as contributed by electromigration can be written as:

For Endothelin

$${\text{J}}_{\text{electromigr}}/\text{Z}=3\text{.49}\times \text{1}{\text{0}}^{-\text{9}}\text{dv}(\text{mol}/\text{s})$$

For Cystatin C

$${\text{J}}_{\text{electromigr}}/\text{Z}=2\text{.58}\times \text{1}{\text{0}}^{-\text{5}}\text{dv}(\text{mol}/\text{s})$$

For Interleukin - 6

$${\text{J}}_{\text{electromigr}}/\text{Z}=6\text{.79}\times \text{1}{\text{0}}^{-\text{11}}\text{dv}(\text{mol}/\text{s})$$

**Figure 6:**
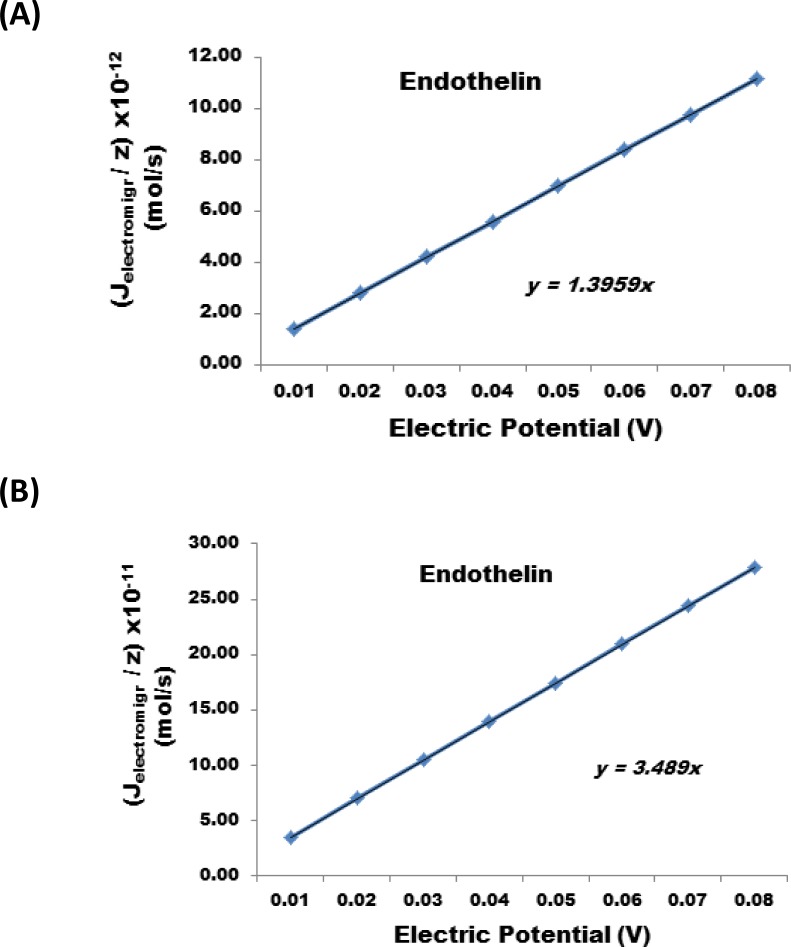
Change in J_electromigr_ for endothelin as a function of applied electric potential passing through a nanofabricated membrane (**A**) 25 m and (**B**) 1 m thick, respectively.

**Figure 7:**
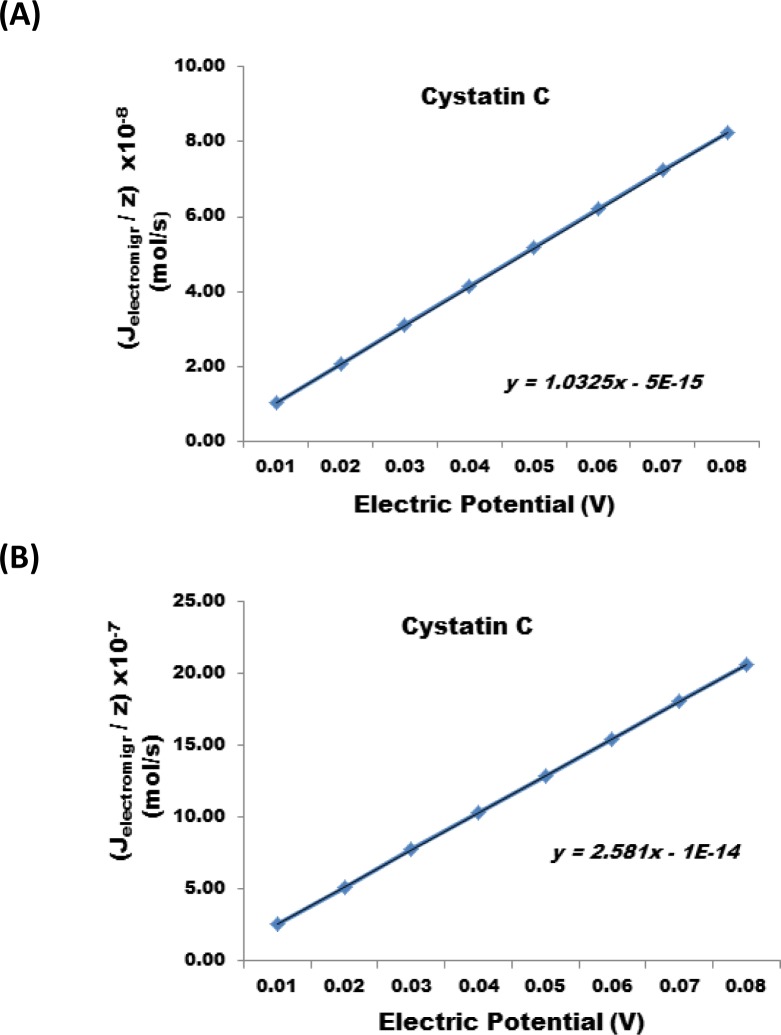
Change in J_electromigr_ for cystatin C as a function of applied electric potential passing through a nanofabricated membrane (**A**) 25 m and (**B**) 1 m thick, respectively.

**Figure 8:**
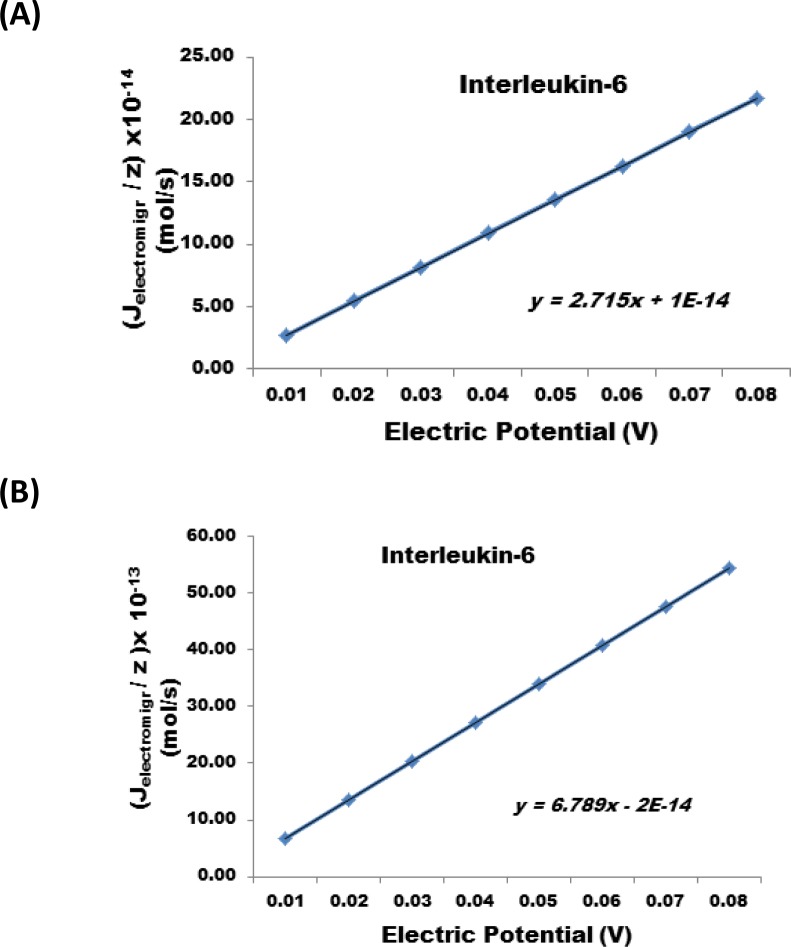
Change in J_electromigr_ for interleukin - 6 as a function of applied electric potential passing through a nanofabricated membrane (**A**) 25 m and (**B**) 1 m thick, respectively.

The results show that a reduction of the nanofabricated membrane from 25 µm to 1 micron at the same applied electric potential can increases the J_electromigr_ by a factor of 25. Figure 6, illustrates the change in J_electromigr_ for endothelin as a function of applied electric potential passing through a nanofabricated membrane 25 µm and 1 micron thick, respectively. Figure 7 illustrates the effect under identical conditions for Cystatin C, and Figure 8 also illustrates the same effect in case interleukin – 6 was transported across the nanofabricated membrane.

As for the calculation of flux change with the increase in pH for the respective molecules passing through a 1 micron thick, nanofabricated membrane, the flux equations could be written as follows:

For Endothelin:

$${\text{J}}_{\text{pH}}=492.30\times 10^{-12}\text{pH}(\text{mol}/\text{s})$$

For Cystatin C:

$${\text{J}}_{\text{pH}}=297.63\times 10^{-8}\text{pH}(\text{mol}/\text{s})$$

For Interleukin - 6:

$${\text{J}}_{\text{pH}}=83.52\times 10^{-14}\text{pH}(\text{mol}/\text{s})$$

**Figure 9:**
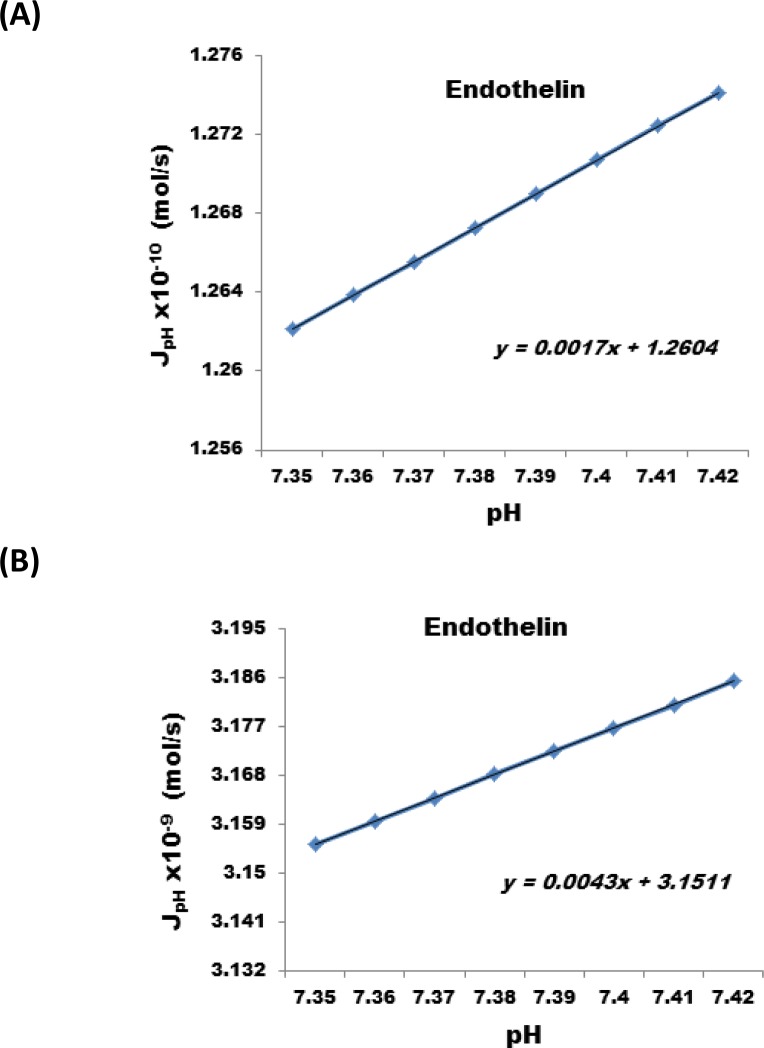
Change in J_pH_ as a function of pH for endothelin passing through a nanofabricated membrane (**A**) 25 m and (**B**) 1 m thick, respectively.

**Figure 10:**
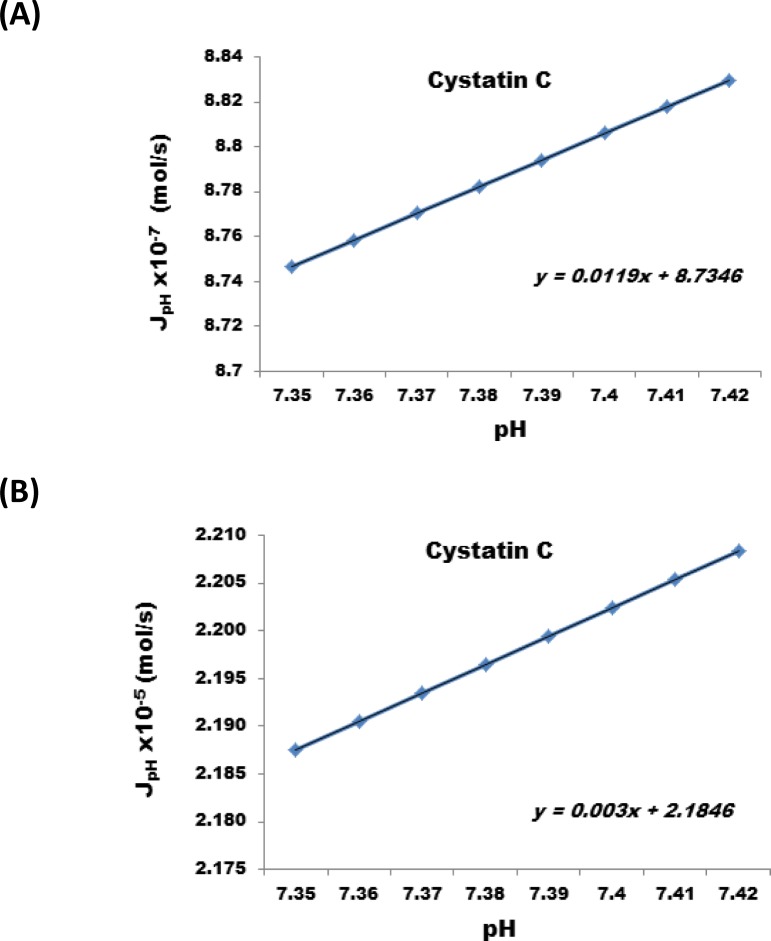
Change in J_pH_ as a function of pH for Cystatin C passing through a nanofabricated membrane (**A**) 25 m and (**B**) 1 m thick, respectively.

**Figure 11:**
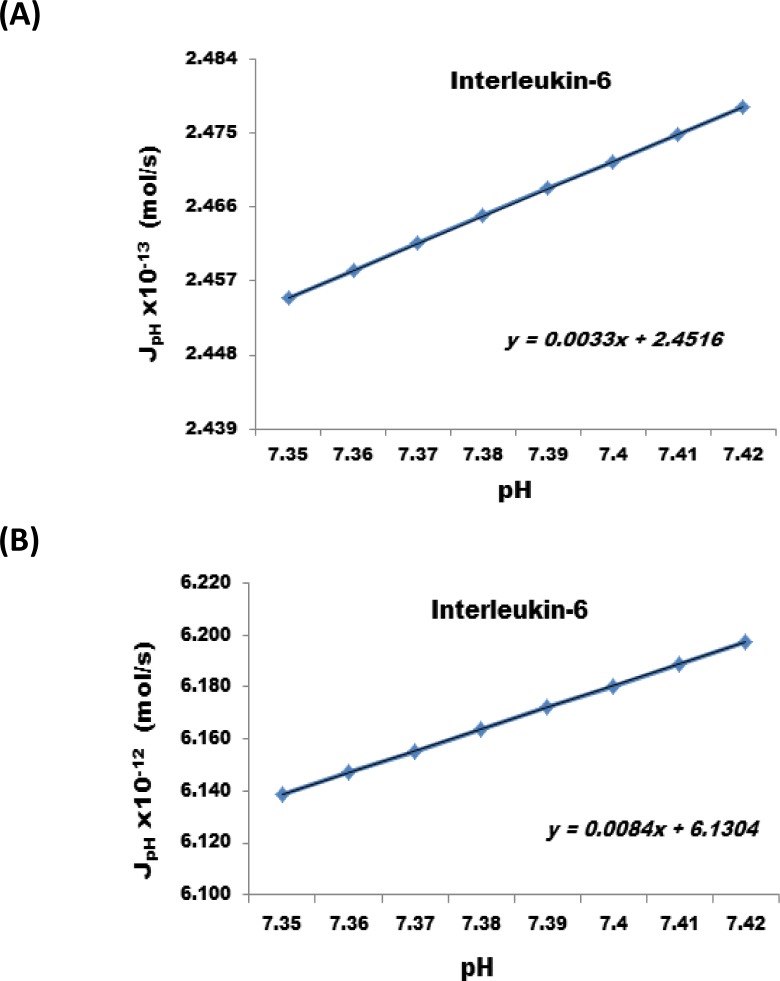
Change in J_pH_ as a function of pH for interleukin - 6 passing through a nanofabricated membrane (**A**) 25 m and (**B**) 1 m thick, respectively.

Figure 9, shows the change in J_pH_ as a function of pH for endothelin passing through a nanofabricated membrane 25 µm and 1 micron thick, respectively. Similarly, Figure 10 illustrates the pH effect on J_pH_ under identical conditions for Cystatin C, and Figure 11 demonstrates the pH effect in case interleukin – 6 was transported across the membrane.

## Discussion

### Morphological characterization of the high flux hemodialyzer

The high flux hemodialyzer's capillaries have different morphologies from the inner, cross-sectional and outer surfaces. As summarized in an earlier study [3], We estimated the pore width to be about 40.11 ± 3.62 nm for a pore width range of 35 nm to 45 nm, with an open pore space in contact with the blood of 5.45 ± 1.41% [3]. The inner pores represent a very low percentage of the overall inner surface, considering the need of higher efficiency to dispose of the uremic toxins. We estimated the pore density to be about 36.81 ± 14.62%, and the pore width to be 11.36 ± 7.80 µm for a pore width range of 0.45 µm to 39.73 µm [3]. Both of the cross-sectional columnar structure and the outer open surface are sufficient for flushing the transported uremic toxins with the dialysate. Due to the large size open pores, we examined the outer open pores looking inwards using both the SEM and the FESEM. The channel structure through which the toxins migrate is definitely tortuous, with a labyrinth of fine ducts that can branch out in various directions. Also, close examinations of cross-section of the capillaries demonstrate the increase in sponginess of the structure from the inner skin of the capillary to the outer surface, as indicated in a previous study [3].

### Determining the functional / structural limitations of current hemodialysis membranes

It is clear from the morphological delineation of the high flux hemodialyzer's capillary that the low pore density on the inner surface represents a structural limitation for the clearance of uremic toxins such as endothelin, cystatin C, and interleukin – 6 through the capillary walls. In addition, the tortuosity of the pores is another limiting factor to the clearance of middle molecules. However, the maximum dimensions of the respective molecules are about one third the width of the fenestrations, which suggests that the pore opening is not a limiting factor to their transport outside the capillary. Possible Causes of Inefficiency of the current HD Sieving Technology are:

- Diminished pore density
- High ratio of the length of the tortuous transport path to the maximum dimension of the uremic toxins molecules
- Significant diffusion and convection hindrances to the transport of uremic toxins through liquid-filled pores, and the interaction between solute molecules and the walls of the pores in the capillaries [17].
- Dis-synergy between diffusion and convection

### Identifying the modifiable sieving parameters that can be improved by nanotechnology to enhance fluxes of uremic toxins across the walls of dialyzers’ capillaries

The pore size density on the inner walls of the capillaries, as well as the channels within the 50 µm thick needs to be significantly improved. Nanfabrication of hemodialysis membranes can overcome these structural limitations present in the high flux hemodialyzer. The membranes could be made thinner, provided that they can withstand the mechanical loading on them. The channels can be structured straight, i.e with a tortuosity equal to 1, and the pattern of the open space can be designed to maximize pore density.

Also, diffusion and convection are the two dominant driving forces in the sieving process of the uremic toxins across the capillary walls. As given by equation (11), the diffusion hindrance increases to the second power with the decrease in open space. Thus, for a structure similar to that of the high flux hemodialyzer with limited open space in the inner surface of the capillary walls, it is expected that the smaller molecules will sieve faster than the middle ones such as endothelin, cystatin C, and interleukin – 6. Convection is the difference in hydraulic pressure across the hollow fiber's walls. We estimated the tension on each hollow fiber's wall to be about 0.066 mm Hg (1.3 x 10^-3^ psi) [18]. This is relatively low given the 50 µm thickness of each capillary.

Inside the pores, there are a number of factors affecting the transport of the molecules. There is steric partitioning taking place where it is more challenging for a larger molecule to fit in a pore than a smaller one. There is also electrostatic partitioning resulting from the Debye repulsion layer that results if both the pore and the molecule are similarly charged. In addition, the molecules are hindered by the adjacent walls, a phenomenon known hydraulic hindrance. Both diffusion and convection of the molecules are affected by steric and hydraulic hindrances [17]. These complex interactions makes us conclude that both diffusion and convection forces are anti-synergistic [4].

### Utilizing irreversible (non-equilibrium) thermodynamics to model the enhancement of uremic toxins transport in nanofabricated hemodialysis membranes

It is clear how IT can be a powerful tool to be used to predict the contribution of the different forces to the overall solute flux across the membrane. In nanofabricated membranes, IT can be implemented to design the channels in the membrane in such a way that the forces influencing the transport are synergistic [4]. Equation (1) predicts the fluxes contributed by diffusion, electro-migration, proton motive force, convection, and ultrafiltration, and sums them together. Thus, through nanofabrication of hemodialysis membranes we can control the channel structure and applied electric current to make all driving forces involved in filtration to act synergistically.

Sieving across the hemodialysis membranes is proportional to their porosity, and the sieving coefficient is inversely proportional to their thickness [19]. Both characteristics are achievable by nanofabrication. Also, we can apply an electric potential on nanofabricated surfaces to enhance the uremic toxin fluxes across them. Uremic toxins have either a net positive or negative charge, or ar neutral. For example, interleukin-6 has both positive and negative surface charges at different molecular sites [20]. It was also reported that some molecules could change shape with the application of an electric field on the membrane's surface [21]. In this study, we found that the application of an electric potential as low as 10 mV can contribute to an increased flux of endothelin, cystatin C, and interleukin – 6. A number of attempts to nanofabricate hemodialysis membranes were made. The methods used, materials, and their drawbacks were the focus of one of our recent publications [22].

In contrast to the significant contribution of the electric gradient to the overall flux enhancement of the respective uremic toxin, the change in pH did not affect their flux significantly. This confirms our findings for the effect of ΔpH on the flux of creatinine, β2-macroglobulin and tumor necrosis factor - α, that we pursued in a previous study.

## Limitations

Our calculations are limited by the fact that many molecules, particularly proteins, may get adsorbed to the membrane, and this represents a form of elimination. In addition, the effects of protein and cell adsorption on the changes in the membrane's characteristics are not addressed by these theoretical calculations. In-vivo experimentation is always needed to comprehensively address these limitations.

## Conclusions

- Nanofabricated membranes designed to have an increased pore density and an ameliorated channel structure can improve the transport of middle molecules across them.
- D_eff_ of Uremic toxins through nanofabricated membranes is 1000 times higher than D_eff_ in the high flux hemodialyzer.
- Theoretical irreversible thermodynamics modeling of uremic toxins fluxes through nanofabricated hemodialysis membranes is a strong tool to predict the contribution of the different forces to the solute flux.
- The driving forces of filtration can act in synergy in a nanofabricated membrane used in hemodialysis.
- Application of an electric potential to the membrane produces an electric driving force that can overwhelm diffusion and convection.
- Thinner nanofabricated membranes improve solute fluxes.
- Slight variation in pH does not affect the flux of uremic toxins significantly.

## Figures and Tables

**Table:** 

H_2_:	Hydrogen molecule
z:	Valence
F:	Faraday's constant
P_H2_:	Partial pressure of hydrogen
R:	Gas constant
D_eff_:	Effective diffusivity
C_m_:	Solute's concentration inside the membrane
A:	Area of the membrane
x:	Thickness of the membrane
D_o_:	Diffusion coefficient
J:	Solute flux
dC/dx:	Concentration gradient across the membrane
T:	Temperature
dV/dx:	Electric potential difference across the membrane
k_diff_:	Diffusion hindrance
k_conv_:	Convection hindrance
J_v_:	Parabolic fluid velocity
J_diff_:	Solute flux by diffusion
J_electromigr_:	Solute flux by electromigration
J_p_:	Solute flux by ultrafiltration
L_p_:	Hydraulic permeability of the membrane for water, that is, the volumetric flow rate of water per unit area of membrane per unit pressure gradient (mL/min/m2 /mmHg)
ΔP:	Hydraulic pressure gradient from blood path to dialysis fluid
Δπ:	Osmotic pressure gradient from blood path to dialysis fluid path
Ω:	Solute concentration parameter
r:	Molecular radius
S:	Sieving coefficient
C_bulk_:	Solute concentration in the bulk plasma water
η:	Viscosity of water at 37°C
V_M_:	Molecular volume
b:	Radius of a cylindrical pore
λ:	Ratio r/b
